# The role of γ-aminobutyric acid in aluminum stress tolerance in a woody plant, *Liriodendron chinense* × *tulipifera*

**DOI:** 10.1038/s41438-021-00517-y

**Published:** 2021-04-01

**Authors:** Pengkai Wang, Yini Dong, Liming Zhu, Zhaodong Hao, LingFeng Hu, Xiangyang Hu, Guibin Wang, Tielong Cheng, Jisen Shi, Jinhui Chen

**Affiliations:** 1 Key Laboratory of Forest Genetics & Biotechnology of Ministry of Education of China, Co-Innovation Center for Sustainable Forestry in Southern China, Nanjing Forestry University, Nanjing, 210037 China; 2 Suzhou Polytechnic Institute of Agriculture, Suzhou, 215008 China; 3 Shanghai Key Laboratory of Bio-Energy Crops, School of Life Sciences, Shanghai University, Shanghai, 200444 China; 4 College of Forestry, Nanjing Forestry University, Nanjing, 210037 China; 5 College of Biology and the Environment, Nanjing Forestry University, Nanjing, 210037 China

**Keywords:** Abiotic, Plant physiology

## Abstract

The aluminum (Al) cation Al^3+^ in acidic soil shows severe rhizotoxicity that inhibits plant growth and development. Most woody plants adapted to acidic soils have evolved specific strategies against Al^3+^ toxicity, but the underlying mechanism remains elusive. The four-carbon amino acid gamma-aminobutyric acid (GABA) has been well studied in mammals as an inhibitory neurotransmitter; GABA also controls many physiological responses during environmental or biotic stress. The woody plant hybrid *Liriodendron* (*L. chinense* × *tulipifera*) is widely cultivated in China as a horticultural tree and provides high-quality timber; studying its adaptation to high Al stress is important for harnessing its ecological and economic potential. Here, we performed quantitative iTRAQ (isobaric tags for relative and absolute quantification) to study how protein expression is altered in hybrid *Liriodendron* leaves subjected to Al stress. Hybrid *Liriodendron* shows differential accumulation of several proteins related to cell wall biosynthesis, sugar and proline metabolism, antioxidant activity, cell autophagy, protein ubiquitination degradation, and anion transport in response to Al damage. We observed that Al stress upregulated glutamate decarboxylase (GAD) and its activity, leading to increased GABA biosynthesis. Additional GABA synergistically increased Al-induced antioxidant enzyme activity to efficiently scavenge ROS, enhanced proline biosynthesis, and upregulated the expression of *MATE1/2*, which subsequently promoted the efflux of citrate for chelation of Al^3+^. We also showed similar effects of GABA on enhanced Al^3+^ tolerance in *Arabidopsis*. Thus, our findings suggest a function of GABA signaling in enhancing hybrid *Liriodendron* tolerance to Al stress through promoting organic acid transport and sustaining the cellular redox and osmotic balance.

## Introduction

Acidic soil occurs frequently on Earth; ~60% of tropical and subtropical areas suffer from soil acidity, which severely limits crop yield. Environmental pollution and acid rain may also contribute to increased soil acidity. The rhizotoxic Al^3+^ ions become soluble in acidic soil with pH values below 5 and dramatically suppress root growth. Thus, Al toxicity has become a serious agronomic problem that restricts crop yield; enhancing the resistance of crops and biofuels to Al will be a valuable strategy to increase their productivity^1–3^. The plant Al resistance mechanism can be divided into external exclusion or internal detoxification based on whether it occurs within or outside of the plant cell. Several mechanisms have been suggested to explain the external exclusion mode of Al resistance^4^. The most well-known strategy is the efflux mechanism of organic acid ions, including citrate, oxalate, or malate, from the root tip, which may then directly chelate external Al to prevent Al toxicity^4–6^. For most crop plants, such as rice and wheat, or model plants, such as *Arabidopsis*, Al toxicity can be a strong growth deterrent; however, most forest trees show a high tolerance to Al stress. For example, Norway spruce (*Picea abies*) or birch (*Betula pendula*) may endure Al concentrations of up to 3 mM in the soil; however, an Al concentration below 50 µM noticeably suppresses root elongation in *Arabidopsis*^7^. Most woody plants grow naturally in acidic soil and have evolved specific mechanisms to cope with high Al stress^8^. Therefore, deciphering the mechanism underlying the tolerance of woody plants to Al stress could help us understand the same process in crops and facilitate the use of gene-engineering strategies to improve Al tolerance in crop plants.

Correspondingly, a series of transporter genes responsible for Al-activated exudation of malate or citrate have been reported. First, *TaALM1* (*Triticum aestivum* Al-activated malate transporter) was identified as a malate anion efflux transporter for Al resistance^9^. In *Arabidopsis* and rape (*Brassica napus*), homologs of *TaALM1* were similarly identified as Al^3+^-enhanced malate transporters^10,11^. Through the map-based cloning method, another gene family for Al resistance, known as MATE (multidrug and toxic compound extrusion) transporters, was identified in sorghum^12^. Several MATE orthologs that function as citrate transporters were then isolated in *Arabidopsis* (*AtMATE1*), maize (*ZmMATE*), rice bean (*VuMATE*), and rice (*OsFRD1*)^13^. More recent evidence showed that the abundance of these transporter families could also be transcriptionally regulated. Two zinc-finger proteins, namely, STOP1 from *Arabidopsis* and ART1 from rice, can directly regulate the Al-induced expression of *ALMT1* and *AtMATE*^14,15^. Furthermore, the WRKY transcription factor WRKY46 can bind the ALMT1 promoter to suppress its expression during Al stress, likely as part of a negative feedback loop^16^. In addition to Al chelation using organic acids, another effective strategy for Al exclusion is the absorption of Al by plant cell wall polysaccharides. At a high Al concentration in the environment, barley (*Hordeum vulgare*) may absorb ~85% of the peripheral Al into its root cell walls^17^, and the giant alga *Chara coralline* may even absorb up to 99.9% of total Al into its cell walls^18^.

The four-carbon amino acid gamma-aminobutyric acid (GABA) functions as an inhibitory neurotransmitter in animals^19^. In plants, GABA was found to regulate the responses to various abiotic stresses, such as heat, cold, touch, or hypoxia stress, as well as biotic stresses, including herbivory, wounding, and pathogen infection. GABA signaling also regulates the balance of C:N or the cytosolic pH^20^. GABA is biosynthesized from glutamate through a Ca^2+^-calmodulin-related enzyme, decarboxylase (GAD), in plants. GABA can also be degraded in mitochondria through the GABA shunt, which contains two continuous steps from the tricarboxylic acid cycle. During the GABA shunt cycle, GABA is converted to succinic semialdehyde by GABA transaminase (GABA-T), and then, succinic semialdehyde is oxidized to succinate by succinic-semialdehyde dehydrogenase (SSADH), coupled with NADH production^19,21^. In *Arabidopsis*, salt stress induces the transcriptional upregulation of GAD and GAD2, resulting in a high level of GABA. Consistent with this, the *pop2* mutant, which is deficient in GABA-T, is very sensitive to ionic stress, such as salt stress, but insensitive to osmotic stress^22^. GABA has also been reported to regulate the malate-transporting plasma membrane channel during Al stress in wheat^23,24^, but the mechanism by which GABA enhances plant tolerance to Al needs to be investigated.

*Liriodendron* is a genus of the magnolia family that has two species, namely, *L. chinense* and *L. tulipifera*. Both of them are widely cultivated horticultural trees in China that produce high-quality timber^25^. Investigating how *Liriodendron* copes with abiotic stress, such as Al toxicity, is therefore of great ecological and economic value. In this study, we studied how hybrid *Liriodendron* responds to Al stress by using a quantitative iTRAQ proteomics approach. Using our available transcriptome data of *L. chinense* as a reference, we successfully isolated 198 proteins that showed significant differential expression after exposure to Al stress; this set included proteins required for energy metabolism, antioxidant activity, and defense response. Among these proteins, we detected upregulation of a putative GAD homologue after Al stress, accompanied by increased accumulation of the AlMT channel protein. The malate content and GAD-dependent GABA content were also increased after Al stress; physiological analysis showed that suppressing GABA biosynthesis aggravated, while application of exogenous GABA attenuated, the Al toxicity-mediated damage to hybrid *Liriodendron* viability. In addition, we found that the role that GABA plays in mediating Al stress resistance is conserved in both poplar and *Arabidopsis*. Thus, our findings suggest a new and conserved mechanism by which GABA enhances the tolerance of hybrid *Liriodendron* to Al stress through AlMT channel-dependent malate efflux.

## Materials and methods

### Plant materials and Al treatment

In this study, plantlets derived from the *L. chinense* somatic embryogenesis system were used as previously reported^26^. In brief, embryogenic calli were induced from immature seeds and grown on the induction medium, and then, the induced calli were moved into liquid culture to promote their growth for 2 or 3 weeks. The embryogenic cells were shifted to solid medium for 4–5 weeks to induce seedling generation. The regenerated seedlings were transplanted to 0.5-L pots with fine soil under greenhouse conditions (relative humidity of 50–70%, 25 °C, white light at 800 µmol photons m^−2^ s^−1^). Each pot was watered with 300 mL of nutrient solution containing KNO_3_ (1 mM), Ca(NO_3_)_2_ (1 mM), KH_2_PO_4_ (0.1 mM), MgSO_4_ (0.5 mM), H_3_BO_3_ (20 μM), MnCl_2_ (2 μM), ZnSO_4_ (2 μM), CuSO_4_ (0.5 μM), (NH_4_)_6_Mo_7_O_24_ (0.065 μM), and Fe-EDTA (20 μM) every 5 days. The nutrient solutions containing the indicated AlCl_3_, GABA or AlCl_3_ + GABA concentration were used for the Al stress treatments.

### Chlorophyll fluorescence analysis

Plant leaves after different treatments were collected for chlorophyll fluorescence intensity analysis using a chlorophyll fluorometer (Heinz Walz GmbH, Effeltrich, Germany)^27^. Each detached leaf was placed in the dark for 30 min for dark adaptation. Then, the maximum quantum yield of PSII was monitored as Fv/Fm. The maximum fluorescence (Fm) was measured with 4000 µmol s^−1^ m^−1^ light with a 0.8-s pulse. Every analysis was repeated at least three times.

### Analysis of the H_2_O_2_ and O_2_^−^ content

The H_2_O_2_ content was determined using xylenol orange^28^. In brief, hybrid *Liriodendron* leaf tissue (1 g) was collected after different treatments and homogenized in 5 mL of HClO_4_ solution (0.2 M) in a cold room at 4 °C. After allowing the mixture to stand for 5 min, the supernatant was obtained after 10 min of centrifugation at 10,000 × *g*, and then, 100 μl of the supernatant was added to 1 mL of reaction buffer to analyze H_2_O_2_ content; the reaction was processed at room temperature for 1 h. The H_2_O_2_ level was measured by calculating the absorbance at 560 nm based on an H_2_O_2_ standard curve.

The O_2_^−^ content was determined according to a previously published method^29^. The supernatant (100 μl) was incubated with 1 mL of the reaction mixture with 50 mM potassium phosphate buffer (pH 7.0), 10 mM hydroxylamine hydrochloride, 17 mM sulfanilic acid, and 7 mM α-naphthyl. The absorbance at 530 nm was monitored, and the O_2_^−^ level was measured based on an O_2_^−^ standard curve.

### Lipid peroxidation analysis

Lipid peroxidation was analyzed using the thiobarbituric acid-reacting substances (TBARS) method^27^. Leaf samples of hybrid *Liriodendron* (1 g) were frozen quickly in liquid nitrogen and homogenized using 10 mL of extraction buffer with trichloroacetic acid (TCA) at 10%. The extract was centrifuged for 20 min at 10,000 × *g*, and then, the supernatant was used for further analysis. One milliliter of supernatant was added to 4 mL of reaction buffer containing 0.6% thiobarbituric acid and 20% TCA, and the mixture was incubated at 95 °C for 30 min. Then, the reaction was stopped by rapid cooling in an ice bath. After the reaction had cooled to room temperature, the mixture was centrifuged at 10,000 × *g* for 10 min, and the absorbance of the supernatant was monitored at 532 and 600 nm. The difference between the absorbance of the supernatant at 532 nm and that at 600 nm was measured, and the MDA level was calculated as previously described^27^.

### RNA extraction and RT-qPCR analysis

For quantitative RT-PCR analysis, hybrid *Liriodendron* leaves after different treatments were used for total RNA extraction using TRIzol reagent (Tiangen, China). The synthesis of first-strand cDNA and quantitative RT-PCR were performed using a previously reported method^28^. The primers used for RT-qPCR are listed in Supplemental Table 2. For each sample, three individual repeats of biological experiments were used for statistical analysis.

### Antioxidant enzyme activity measurements

Leaf tissue (~1 g) was collected for enzyme activity analysis. Different antioxidant enzyme activities, including ascorbate peroxidase (APX), glutathione reductase (GR), monodehydroascorbate reductase (MDHAR), and dehydroascorbate reductase (DHAR) activities, were analyzed as previously described^27^. The protein content was calculated using the Bradford method^29^.

### Analysis of GABA content and GAD enzyme activity

Measurement of the GABA content in leaf extracts was performed as previously described^30^. In brief, ~10 g of leaf sample was powdered in a Falcon tube and extracted using 10 mL of 80% (v/v) ethanol. The extraction buffer was collected and centrifuged for 10 min at 1200 × *g* and 4 °C, and the supernatant was removed and filtered using Millipore filter paper. The filtration was repeated three times, and the filtrates were combined and dried on a rotary evaporator until the ethanol completely evaporated. Then, the dried residue was dissolved in 1 mL of water, and 1 mL of methanol containing 2-hydroxynaphthaldehyde (2.5% w/v) was added to the dissolved GABA for derivatization. Then, 0.5 mL of boric acid-NaOH (pH 8.5) was added to neutralize the solution. The resultant sample was dried for 20 min at 85 °C and cooled to room temperature. The residue was dissolved in 5 mL of methanol for further analysis. HPLC analysis was performed using an Agilent 1200 HPLC instrument. Approximately 5 µl of the solution was injected onto a reversed-phase SB-C18 column with a methanol gradient system (2 min of 60% methanol, 5 min of 70% methanol, 8 min of 80% methanol, 10 min of 90% methanol, and 12 min of 50% methanol) with a flow rate of 0.8 mL/min. A UV detector at 254 nm was used to monitor the GABA content. The GABA content was analyzed by comparison with the retention time of a standard.

GAD activity was calculated by measuring the conversion ratio of the substrate glutamate to GABA^22^. Approximately 1 g of plant leaf sample was extracted in 10 mL of reaction buffer containing 80 mM sodium phosphate (pH 5.6) and 100 mM L-glutamate and then kept for 60 min at 40 °C. Then, the reaction was stopped at 90 °C. The reaction was cooled and centrifuged for 10 min at 1200 × *g*, and then, the supernatant was taken for GAD enzyme activity analysis as reported previously^22^.

### Proline content measurement

Pro accumulation in hybrid *Liriodendron* leaves was measured using a previously described method^27^, using l-Pro as a standard. In brief, ~1 g of leaves was collected and extracted using 3% sulfosalicylic acid in a cold room. The supernatant was obtained after centrifugation at 12,000 × *g* for 10 min at 4 °C. An aliquot (2 mL) of supernatant and ninhydrin solution containing 2.5% [w/v] ninhydrin, 40% 6 M phosphoric acid, and 60% [v/v] glacial acetic acid was reacted at 100 °C for 30 min, and the reaction was stopped by adding ice for quick cooling. Then, 5 mL of toluene was added, and the solution was incubated at room temperature overnight. The proline content was monitored by measuring the absorbance at 520 nm using a spectrophotometer.

### Relative root growth (RRG) analysis

RRG values were determined as previously reported^2^. The seeds were surface-sterilized and sown in Murashige and Skoog (MS) medium for 3 days under light conditions at 60 µmol photons m^−2^ s^−1^, and the initial root length was measured. Then, half of the seedlings were shifted to MS medium containing different concentrations of AlCl_3_, while the remaining half of the seedlings continued to grow on the same MS medium without Al stress. After 5 days of growth under the same light conditions, the root length was monitored using a ruler, and the degree of inhibition of root elongation is presented as the percentage of RRG.

### Total leaf protein extraction

Total leaf proteins were extracted from frozen samples using phenol extraction buffer as previously described^31^. Briefly, ~10 g of leaf tissue was quickly frozen and ground in liquid nitrogen using a mortar and pestle, followed by extraction in 10 mL of ice-cold protein extraction buffer (100 mM Tris-HCl buffer (pH 7.8), 100 mM KCl, 1% v/v Triton X-100, 1% v/v β-mercaptoethanol, 50 mM L-ascorbic acid, 1 mM phenylmethanesulfonyl fluoride). The supernatant was obtained by centrifugation at 12,000 × *g* for 10 min and mixed with an equal volume of Tris-phenol buffer (100 mM Tris, pH 8.0). The mixture was vortexed thoroughly, and the upper phenol phase was collected after centrifugation at 12,000 × *g* at 4 °C for 30 min. Finally, five volumes of methanol containing 10 mM ammonium acetate were added to the upper phenol phase. The mixture was placed at −20 °C overnight, and the protein pellet was obtained by centrifugation at 12,000 × *g* for 15 min. The wet pellet was washed with cold acetone and 0.1% β-mercaptoethanol three times, and the washed pellet was then dried in air. The dried pellet was stored at −80 °C for further use or dissolved in Tris-HCl buffer (pH 8.5, 40 mM) containing urea (7 M), EDTA (2 mM), thiourea (2 M), CHAPS (4% v/v), and PMSF (1 mM) at a final concentration of 10 mg/mL for the next proteomic analysis. The protein solution was sonicated at 200 W for 15 min to promote dissolution and then centrifuged at 12,000 × *g* for 15 min at 4 °C. The pellet was discarded, and the supernatant was transferred to another tube. Then, 10 mM DTT was added to the protein solution to avoid disulfide bond formation. Iodoacetamide (IAM) was added at 55 mM to the protein solution under dark conditions to covalently block cysteines. Finally, the protein pellet was obtained by adding 5 volumes of cold acetone at −20 °C for 2 h. The pellet was dried again and dissolved using 500 μl of TEAB (tetraethylammonium bromide, 0.5 M), and the supernatant was collected after centrifugation at 12,000 × *g* for 15 min in a cold room for proteomic analysis. The protein concentration was quantified using the Bradford method and a Bio-Rad protein assay kit (Bio-Rad, USA).

### iTRAQ mass spectrometry analysis

The protein (100 µg) extracted from the leaves as described above was used for iTRAQ analysis as follows. Briefly, the extracted protein was first digested to peptides by Gold Trypsin (Promega, Madison, WI, USA) at a ratio of 30:1 (protein:trypsin) at room temperature for 16 h and then dried by vacuum centrifugation. The dried peptides were then redissolved in TEAB buffer (0.5 M) as recommended for the 8-plex iTRAQ reagents (AB Sciex Inc., MA, USA). The extracted protein from different samples was digested as described above and labeled with iTRAQ reagents with the 113 to 117 isobaric tags at room temperature for 2 h. After labeling, the peptide mixture was dried through vacuum centrifugation and redissolved in 4 mL of strong cation exchange (SCX) solvent containing 25 mM NaH_2_PO_4_, 25% acetonitrile, and 10 mM ammonium formate (pH 2.7). The peptide mixtures were separated by an Ultremex SCX column (4.6 × 250 mm) using a Shimadzu LC-20AB HPLC pump system (Shimadzu Co., Kyoto, Japan). The peptide fraction was eluted at a flow rate of 1 mL min^−1^ with stable 5% buffer B containing 1 M KCl, 25 mM NaH_2_PO_4_, and 25% ACN at pH 2.7 for 7 min, followed by a linear gradient of 5–60% buffer B over 20 min and 60–100% buffer B over 2 min. Finally, elution was performed with 100% buffer B. The absorbance at 214 nm was selected to monitor the elution peak, and a total of 20 fractions were collected. Each SCX fraction was desalted and redissolved in buffer C (5% ACN and 0.1% formic acid), and the supernatant was collected by centrifugation at 20,000 × *g* for 10 min. Finally, 5 μL of supernatant was loaded for HPLC-mass spectrometry analysis by Shimadzu LC-20 AD nanoHPLC (Shimadzu Co. Kyoto, Japan) with a C18 column (200 µm inner diameter), and the peptide was eluted with 5% buffer D (95% ACN, 0.1% formic acid) for 5 min, followed by 3–35% buffer D over 35 min, 60–80% buffer D over 2 min, and a final wash with 80% buffer D at a flow rate of 250 nL/min. The eluted peptides were then sprayed into the injection port of a TripleTOF 5600 system (AB SCIEX, Concord, ON, Canada) equipped with a Nanospray III source under a spray voltage of 2.5 kV, 30 psi N gas, 15 psi nebulizer gas, and a heater temperature of 150 °C. Full-scan mass spectra were obtained by the Orbitrap analyzer in information-dependent acquisition (IDA) mode with a mass range of 100–2400 *m/z* at a high detection resolution of over 30,000 (FWHM). The 30 most intense precursor ion peaks with a threshold over 120 cps and a 2+ to 5+ charge state were selected for collision-induced fragmentation. Dynamic exclusion was employed within 40 s to prevent repetitive selection of the peptides.

### Bioinformatics analysis

The raw LC-MS/MS data files were converted to generic Mascot files (mgf) by Proteome Discoverer 1.2 software, and searching was performed with the *L. chinense* proteome database^25^. Trypsin was used as the proteolytic enzyme, and one missed cleavage was permitted. The peptide mass and fragment mass tolerance values were 10 ppm and 0.1 Da, respectively. The search parameters included iTRAQ 8-plex quantification, oxidation of methionine residues, and pyroglutamate formation of N-terminal glutamine residues as variable residues and carbamidomethyl formation of cysteine residues as fixed modifications. Only the peptides with a significant score over 20 with a 99% confidence interval and false discovery rates (FDR) <1.5% were used for quantification analysis, and cutoffs higher than 1.5- or lower than 0.6-fold with a *p*-value < 0.05 were identified as significantly differentially expressed proteins.

## Results

### Al stress impedes photosynthesis in hybrid *Liriodendron* and causes membrane lipid oxidative damage

To understand the mechanism underlying the hybrid *Liriodendron* AlCl_3_ stress response, we first aimed to optimize our experimental conditions. Plant photosynthesis efficiency is a good way to estimate physiological plant health in response to various stressors. We therefore determined how AlCl_3_ toxicity affects leaf photosynthesis by examining the dose-dependent response of the Fv/Fm ratio to increasing AlCl_3_ concentrations. The Fv/Fm ratio is an efficient indicator of a leaf’s photosynthetic ability. We found that treating hybrid *Liriodendron* with AlCl_3_ concentrations from 5 to 50 μM gradually reduced the Fv/Fm ratio: once the AlCl_3_ concentration reached 100 μM, the Fv/Fm ratio decreased rapidly (Supplemental Fig. 1). Based on our dose-response curve, we chose to use an AlCl_3_ concentration of 30 μM for further experiments in this study.

**Fig. 1:**
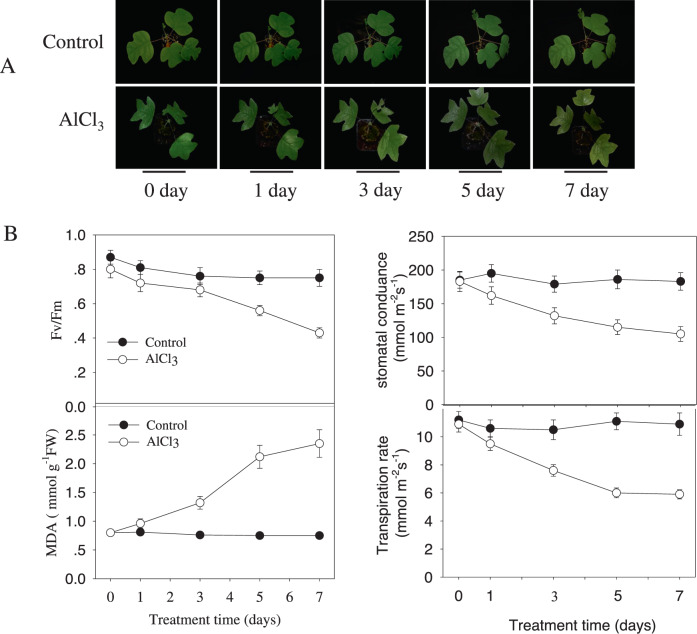
Al stress impedes somatic embryo growth of hybrid *Liriodendron* seedlings. **A** Growth phenotype of hybrid *Liriodendron* in response to Al toxicity. One-week-old seedlings were treated with AlCl_3_ at 30 μM for the indicated times, and photographs were taken. The experiment was performed in triplicate with similar results, and one group of photos is presented. **B** AlCl_3_ inhibits leaf growth-related parameters, including Fv/Fm, MDA, stomatal conduction, and transpiration. One-week-old seedlings were treated with AlCl_3_ at 30 μM for the indicated time, and these growth-related parameters were measured. The experiment was performed in triplicate, and the plotted values are the means ± SDs of three biological replicates

We then proceeded to further characterize hybrid *Liriodendron* leaf viability/photosynthetic capability in response to 30 μM AlCl_3_. We found that hybrid *Liriodendron* leaves exposed to AlCl_3_ turned yellowish after 7 days of treatment, while the Fv/Fm ratio decreased gradually during this time and decreased from ~0.803 to ~0.427. In control plants, the Fv/Fm ratio was stably maintained at ~0.79 (Fig. 1A, B). Lipid peroxidation is generally used to assess the intensity of oxidative stress. Lipid peroxidation generates several reactive aldehydes, such as malondialdehyde (MDA); thus, the level of MDA is used to evaluate environmental oxidative stress. AlCl_3_ treatment also increased the level of MDA, reflecting membrane lipid oxidative damage. Furthermore, we found that the leaf transpiration rate and stomatal conductance levels, additional parameters that reflect photosynthetic activity, were also decreased after AlCl_3_ treatment (Fig. 1B). Thus, these data suggest that AlCl_3_ negatively affected the leaf viability and photosynthetic capability of the *Liriodendron* hybrid.

### Dynamic proteome profiling of hybrid *Liriodendron* in response to Al stress

**Fig. 2:**
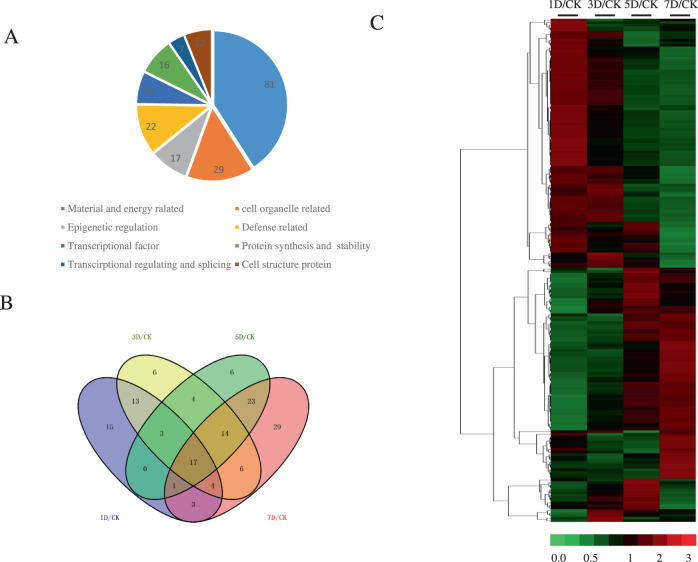
Categorization of differentially expressed proteins in hybrid *Liriodendron* after AlCl_3_ stress. **A** Functional classification of proteins that show differential accumulation under AlCl_3_ stress. **B** Venn diagram analysis of the differentially accumulated proteins after 30 μM AlCl_3_ stress; 1 day/control (1D/CK) indicated the differential protein number after 1 day of AlCl_3_ treatment compared with that under the control condition; 3 day/control (3D/CK) indicates the differential protein number after 3 days of AlCl_3_ treatment compared with that under the control condition; 5 day/control (5D/CK) indicates the differential protein number after 5 days of AlCl_3_ treatment compared with that under the control condition; 7 day/control (7D/CK) indicates the differential protein number after 7 days of AlCl_3_ treatment compared with that under the control condition. **C** Heat-map clustering of the leaf protein abundance profile under AlCl_3_ stress. One-week-old hybrid *Liriodendron* seedlings of somatic embryos were treated with AlCl_3_ stress for 1, 3, and 7 days. Th seedlings without AlCl_3_ stress were used as controls. The protein abundance differences between the treatment and control samples were compared by iTRAQ, and different colors represent the differential abundance ratios of proteins between the treatment and control, as depicted in the bar at the bottom of the figure

Next, we aimed to identify the genes are associated with the Al stress response in hybrid *Liriodendron*. To this end, we performed a proteomics study by using the iTRAQ proteomics approach, which allowed us to analyze differential protein abundance in hybrid *Liriodendron* leaves subjected to 30 μM AlCl_3_ treatment for 1, 3, 5, and 7 days, using untreated plants as a control. Each sample for iTRAQ analysis was repeated with three biological replicates, and protein prediction and quantification were performed through Mascot software and public plant protein databases, as well as our own *L. chinense* transcriptome. Differentially abundant proteins were divided into three groups: group 1 (1 day of AlCl_3_ treatment/untreated control), group 2 (3 days of AlCl_3_ treatment/untreated control) and group 3 (5 days of AlCl_3_ treatment/untreated control). Proteins with a >1.5-fold change in abundance were regarded as having significantly increased expression; in contrast, the proteins with a <0.6-fold change in abundance were regarded as having significantly decreased expression. In total, we identified 198 proteins that presented significant changes after AlCl_3_ treatment (Fig. 2, Supplemental Tables 1 and 2). These 198 identified proteins were classified into 8 groups based on their biological function; most proteins belonged to the largest group of material and energy metabolism, followed by the group of plant hormone signaling and antioxidant proteins. Out of the 198 differentially regulated proteins, 39 proteins with increased expression and 50 proteins with decreased expression were constitutively upregulated or downregulated, respectively, at all time points.

We also performed a hierarchical cluster analysis to identify the proteins that were differentially expressed during Al stress. We noticed that the accumulation levels of antioxidant proteins, such as glutathione S-transferase (Lchi06330), monodehydrogenase reductase (Lchi27219), polyphenol oxidase (Lchi16057), peroxidase 17 (Lchi20649), peroxidase 72 (Lchi10272), and catalase isozyme 1 (Lchi14208), were upregulated. Several proteins associated with protein synthesis or stability, such as proteasome subunit alpha type-1 B (Lchi26499), plant UBX domain-containing protein 4 (Lchi21723), aspartyl protease family protein (Lchi27243), and COP9 signalosome complex subunit 7 (Lchi31560), and proteins associated with epigenetic regulation, such as histone H2A (Lchi09914), thioredoxin M-type (Lchi12001), pre-mRNA-processing protein 40 A (Lchi23330), DEAD-box ATP-dependent RNA helicase 53 (Lchi32037), and putative DNA repair protein RAD23-3 (Lchi24553), were also differentially accumulated after Al stress. In addition, a series of transcription factors, such as zinc finger CCCH domain-containing protein (Lchi14090, Lchi14538), WRKY protein (Lchi09560, Lchi03796, Lchi13688), bZIP transcription factor (Lchi10492, Lchi02202, Lchi09267), and MYB transcription factor (Lchi16241), were also differentially regulated after Al stress.

Organic acids have been reported to function as Al chelators when transported outside the cell, aiding Al toxicity avoidance^4^. Consistent with this, we found several proteins encoding homologs of malate acid transporters, namely, MATE1 (Lchi06125) and MATE2 (Lchi26133), that were upregulated by Al stress, indicating that they may be involved in enhancing the tolerance of hybrid *Liriodendron* to Al stress.

In *Arabidopsis thaliana*, STOP1 belongs to the nuclear zinc finger protein family; it can directly activate AtALMT1 or MATE expression and is involved in attenuating H^+^ and Al^3+^ rhizotoxicity^14^. Our data also showed the upregulation of a zinc finger protein homologous to STOP1 (Lchi25591) in response to Al stress, suggesting a possible common mechanism between hybrid *Liriodendron* and other plants. These results indicate that our proteomics data indeed accurately reflect the translational response of hybrid *Liriodendron* to Al stress.

### Al treatment causes an increase in GAD enzyme activity and GABA synthesis

In addition to proteins that have previously been implicated in the Al stress response, we also detected changes in the expression levels of homologs of GAD and succinic-semialdehyde dehydrogenase (SSADH), both of which are enzymes involved in the GABA biosynthesis pathway in plants^32^. GABA may act as a signaling molecule that is involved in different physiological processes, including growth, development, and defense responses. Our iTRAQ results showed that the abundance of two GAD homologs (Lchi33118 and Lchi05759) and one SSADH homolog (Lchi21261) was upregulated after 1 day or 3 days of Al stress treatment (Supplemental Table 1), suggesting a role for GABA signaling in the hybrid *Liriodendron* Al stress response.

**Fig. 3:**
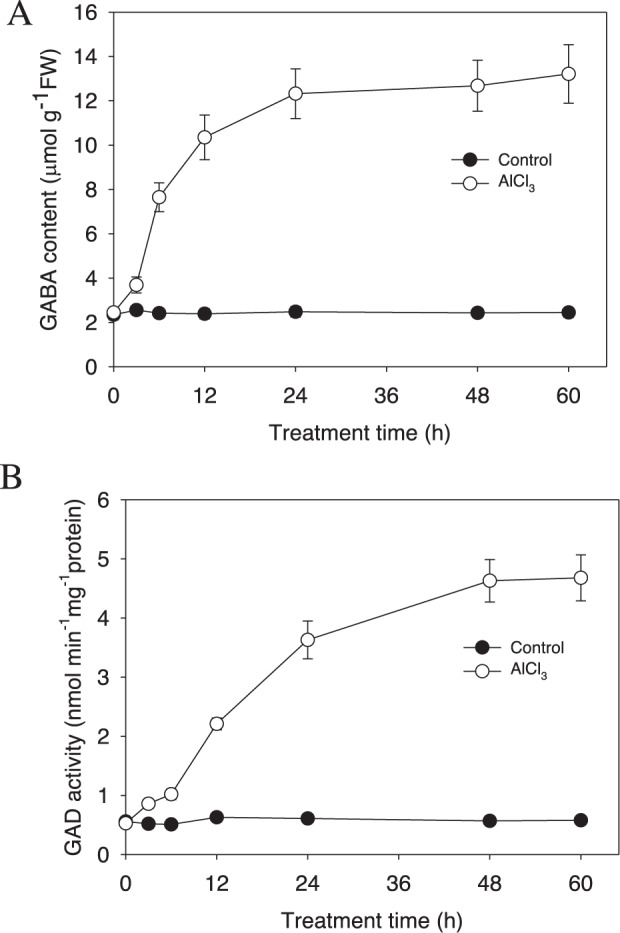
AlCl_3_ stress induces an increase in GABA content and GAD activity. One-week-old hybrid *Liriodendron* seedlings of somatic embryos were subjected to 30 μM AlCl_3_ stress for the indicated amount of time, after which the GABA content (**A**) and GAD activity (**B**) were measured. The experiment was performed in triplicate. The data represent the mean ± SD of three biological replicates

To verify this hypothesis, we determined the GABA level in hybrid *Liriodendron* leaves after exposure to Al stress. AlCl_3_ treatment at 30 μM induced a rapid increase in GABA content in the hybrid *Liriodendron* leaves, with the value reaching 10.35 μmol g^−1^ FW after 24 h of treatment, a high level that was sustained during the following 36 h of treatment (Fig. 3A). In accordance with these data, we also found that GAD enzyme activity (responsible for GABA biosynthesis) increased similarly after Al stress. Al-induced GAD activity peaked after 48 h of Al stress and maintained a high level until 60 h of AlCl3 treatment (Fig. 3B). These data support our proteomics data showing that Al stress induces an increase in GAD protein abundance and indicates a potential role for GABA in the Al stress response in hybrid *Liriodendron*.

### GABA protects hybrid *Liriodendron* from oxidative damage under Al stress

**Fig. 4:**
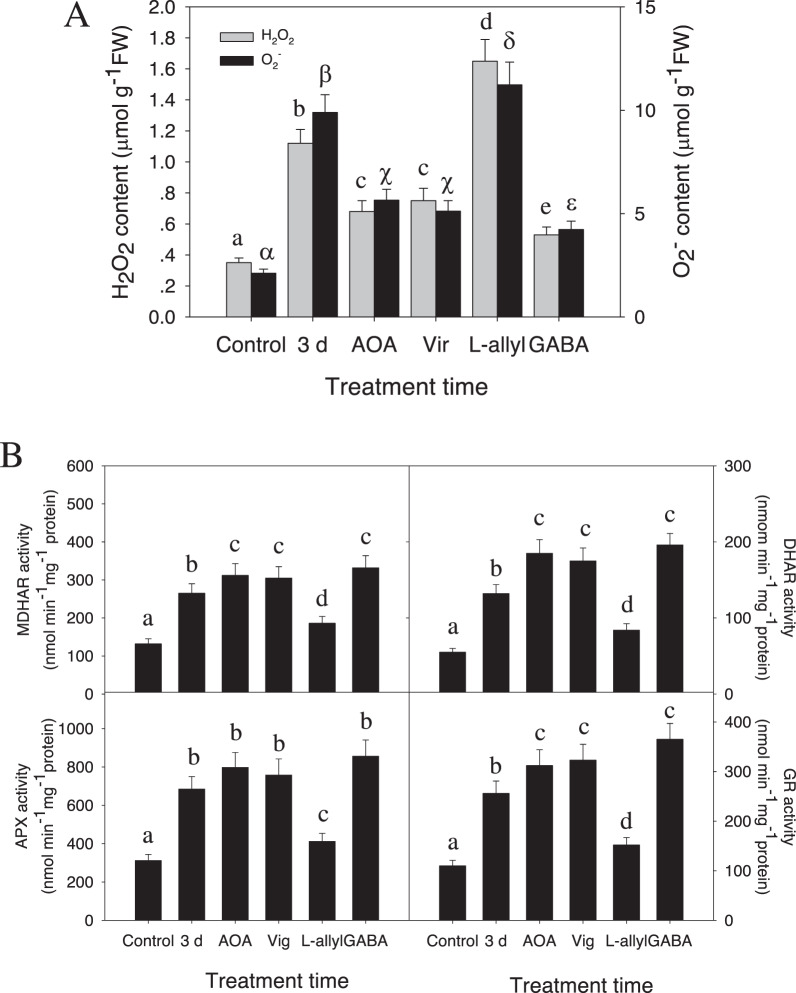
AlCl_3_ stress induces an increase in ROS content and antioxidant enzyme activity. One-week-old hybrid *Liriodendron* seedlings of somatic embryos were subjected to AlCl_3_ stress or AlCl_3_ stress with additional aminooxyacetic acid (AOA), vigabatrin (Vir), L-allylglycine (L-allyl), or GABA for 3 days. Then, the ROS content, including H_2_O_2_ and O_2_^-^ (**A**), and antioxidant enzyme activities, including MDHAR, DHAR, APX and GR activities (**B**), were measured. Control: the sample without Al stress was used as the control; 3 d: 30 μM AlCl_3_ stress for 3 days; AOA: 30 μM AlCl_3_ stress with the addition of 1 mM aminooxyacetic acid for 3 days; Vir: 30 μM AlCl_3_ stress with the addition of 100 μM vigabatrin for 3 days; L-allyl: 30 μM AlCl_3_ stress with the addition of 1 mM L-allylglycine for 3 days; GABA: 30 μM AlCl_3_ stress with the addition of 10 mM GABA for 3 days. The experiment was performed in triplicate, and the data represent the mean ± SD of three biological replicates. Different letters indicate statistically significant differences (*p* < 0.05) as determined by Tukey’s multiple comparisons test

Our above iTRAQ results demonstrate that Al stress increases the expression of proteins encoding antioxidant enzymes, including catalase (Lchi14208), peroxidase (Lchi17145, Lchi20649, Lchi10272) and MDHAR (Lchi27219), which might have a role in scavenging ROS, such as H_2_O_2_ and O_2_^−^, which may accumulate in response to Al stress. To determine whether the Al stress response indeed leads to ROS production, we measured Al-induced accumulation of ROS, focusing on H_2_O_2_ and O_2_^−^. We found that Al stress applied for 3 days induced rapid accumulation of H_2_O_2_ and O_2_^−^ and that pretreatment with exogenous GABA could relieve Al-induced ROS generation (Fig. 4A). These results show that GABA plays a role in scavenging ROS in hybrid *Liriodendron*, which is also consistent with previous studies in plants.

To corroborate these results, we used chemical inhibitors that specifically act to alter the intracellular GABA concentration. Aminooxyacetic acid (AOA) and vigabatrin are putative GABA transaminase inhibitors and suppress the conversion of GABA to SSDHA, thus causing an increase in the endogenous GABA content. L-allylglycine is a GADase inhibitor that suppresses the generation of GABA. Pretreatment with AOA or vigabatrin counteracted AlCl_3_-induced ROS accumulation (Fig. 4A) and enhanced the activities of antioxidant enzymes. This is consistent with an increase in GABA levels, while L-allylglycine treatment increased AlCl_3_-induced ROS damage and reduced antioxidant enzyme activities (Fig. 4B). Similar to AOA or vigabatrin, additional GABA attenuated the AlCl_3_-induced increase in antioxidant enzyme activities (Fig. 4B), suggesting a putative role for GABA signaling in protecting hybrid *Liriodendron* from AlCl_3_-induced oxidative damage.

### GABA enhances proline biosynthesis during Al stress

**Fig. 5:**
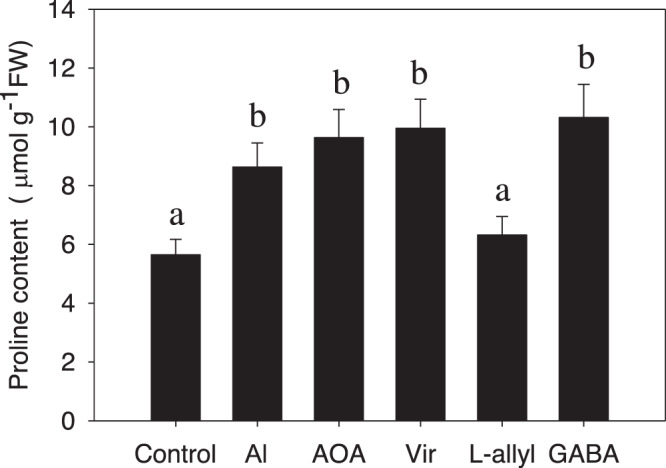
AlCl_3_ stress induces intracellular proline accumulation. One-week-old hybrid *Liriodendron* seedlings of somatic embryos were subjected to AlCl_3_ stress (3 d) or AlCl_3_ stress with the addition of AOA, vigabatrin (Vir), L-allylglycine, or GABA for the indicated amount of time, after which the proline content was measured. The experiment was performed in triplicate, and the data represent the mean ± SD of three biological replicates. Different letters indicate statistically significant differences (*p* < 0.05) as determined by Tukey’s multiple comparisons test

Proline contributes to the plant environmental stress response by altering cellular osmotic levels^33^. Proline-5-carboxylate synthase (P5CS) and proline-5-carboxylate reductase (P5CR) are two essential enzymes that catalyze proline biosynthesis in plants^34^. We found that both the protein levels of P5CS (Lchi29824) and P5CR (Lchi04198) were significantly upregulated in hybrid *Liriodendron* in response to Al stress, suggesting that they may play a role in Al stress tolerance. To study this hypothesis, we monitored the proline level in hybrid *Liriodendron* subjected to Al stress. Al stress induced a marked increase in proline levels starting from 3 days after treatment, after which the levels slowly declined again (Fig. 5). Additional GABA treatment increased Al-induced proline accumulation, which was maintained after 3 to 5 days of Al stress. Furthermore, treatment with the inhibitor AOA or vigabatrin or adding GABA directly strongly increased AlCl_3_-induced proline biosynthesis, while additional L-allylglycine treatment reduced AlCl_3_-induced proline biosynthesis, suggesting a novel role of GABA in proline biosynthesis during Al stress (Fig. 5).

### GABA increases citrate synthesis to combat Al toxicity

**Fig. 6:**
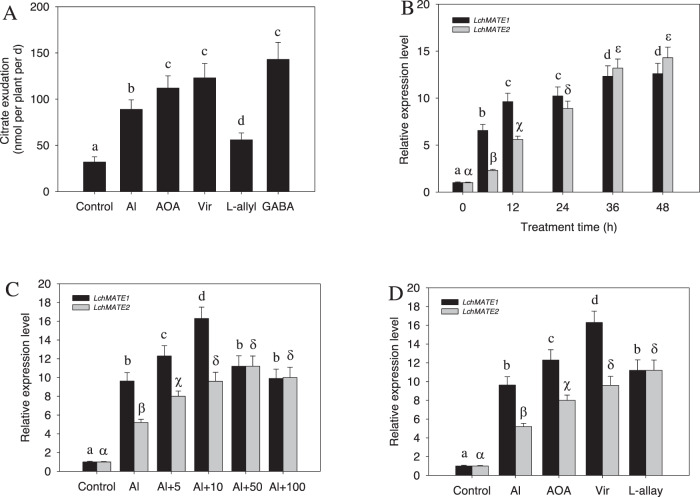
GABA mediates increased *LchMATE* transcription and citrate exudation in response to AlCl_3_ stress in hybrid *Liriodendron* seedlings of somatic embryos. One-week-old hybrid *Liriodendron* seedlings of somatic embryos were subjected to AlCl_3_ stress or AlCl_3_ stress with the addition of AOA, vigabatrin (Vir), L-allylglycine (L-allyl), or GABA, after which citrate exudation and *LchMATE1/2* transcription were measured. The experiment was performed in triplicate, and the data represent the mean ± SD of three biological replicates. Different letters indicate statistically significant differences (*p* < 0.05) as determined by Tukey’s multiple comparisons test. **A** The effects of GABA or different inhibitors on citrate exudation after 3 days of treatment. One-week-old hybrid *Liriodendron* seedlings of somatic embryos were subjected to 30 μM AlCl_3_ stress or 30 μM AlCl_3_ stress with the addition of different inhibitors or GABA, respectively, for 3 days, and citrate exudation was measured. **B** AlCl_3_ stress induced the transcription of *LchMATE1/2*. One-week-old hybrid *Liriodendron* seedlings of somatic embryos were subjected to 30 μM AlCl_3_ stress for the indicated times, and the transcriptional level of *LchMATE1/2* was measured by RT-qPCR analysis. **C** Effect of the GABA concentration on the Al-induced increase in *LchMATE1/2* transcription. One-week-old hybrid *Liriodendron* seedlings of somatic embryos were treated with 30 μM AlCl_3_ or 30 μM AlCl_3_ supplemented with different concentrations of GABA for 24 h, and the transcriptional level of *LchMATE1/2* was measured by RT-qPCR analysis. Control: the sample without Al stress; Al: 30 μM AlCl_3_ treatment; Al + 5: 30 μM AlCl_3_ with an additional 5 mM GABA; Al + 10: 30 μM AlCl_3_ with an additional 10 mM GABA; Al + 50: 30 μM AlCl_3_ with an additional 50 mM GABA; Al+100: 30 μM AlCl_3_ with an additional 100 mM GABA. **D** Effect of different inhibitors on the Al-induced transcriptional increase in *LchMATE1/2*. One-week-old hybrid *Liriodendron* seedlings of somatic embryos were subjected to 30 μM AlCl_3_ stress or 30 μM AlCl_3_ with the addition of different inhibitors as described above for 24 h, and the transcriptional level of *LchMATE1/2* was measured by RT-qPCR analysis

Al treatment may trigger ALMT activity, leading to extracellular transport of malate or citrate and chelation of Al ions. Previous studies found that ALMT is responsible for malate exudation, while MATE exports citrate in most plants, such as *Arabidopsis*, rice and wheat^35^. We found that Al treatment indeed increased the accumulation of MATE1 (Lchi06125, named LchMATE1) and MATE2 (Lchi26133, named LchMATE2) homologs in hybrid *Liriodendron* (Supplemental Table 1 and Supplemental Fig. 2), suggesting a possible function of citrate exudation in hybrid *Liriodendron* Al stress tolerance. We next sought to confirm the functionality of LchMATE upregulation by measuring the extracellular citrate content in hybrid *Liriodendron* in response to Al stress. AlCl_3_ treatment induced a strong increase in citrate, a response that could be further enhanced through additional GABA treatment (Fig. 6A, B). Pretreatment with the enzyme inhibitors AOA and vigabatrin similarly increased citrate exudation, while L-allylglycine treatment reduced citrate exudation, suggesting that GABA signaling additionally controls organic acid-mediated chelation of Al as a stress response. We also investigated the change in malate in hybrid *Liriodendron* after AlCl_3_ or GABA treatment. Although AlCl_3_ treatment obviously increased the malate content, additional GABA treatment did not further increase the malate content (Supplemental Fig. 3), suggesting that malate metabolism is not the main pathway by which GABA protects against AlCl_3_ stress.

Next, we sought to determine whether regulation of *LchMATE1* or *LchMATE2* in response to AlCl_3_ stress occurs solely at the translation level or whether transcriptional regulation is also involved. Through RT-qPCR experiments, we found that AlCl_3_ induces a dose-dependent transcriptional response in *LchMATE1* or *LchMATE2*, with GABA synergistically further increasing the AlCl_3_-induced transcriptional levels of *LchMATE1/2* (Fig. 6C). Consistent with these results, AOA and vigabatrin treatment also coordinately increased AlCl_3_-induced citrate exudation, while L-allylglycine treatment exerted the opposite effect (Fig. 6D). There are two isomers of GABA in plants, namely, alpha-aminobutyric acid (AABA) and beta-aminobutyric acid (BABA). BABA has been indicated to enhance plant resistance to disease and abiotic stress. Many studies have shown the difference between BABA and GABA^36,37^. As an isomer of GABA, AABA is not well studies. The hybrid *Liriodendron* was further treated with AlCl_3_, GABA, and AABA to determine whether regulation of *LchMATE1* or *LchMATE2* was performed by this analog of GABA. The RT-qPCR data show that the transcription of *LchMATE1* or *LchMATE2* could hardly be regulated by AABA in hybrid *Liriodendron* (Supplemental Fig. 4).

**Fig. 7:**
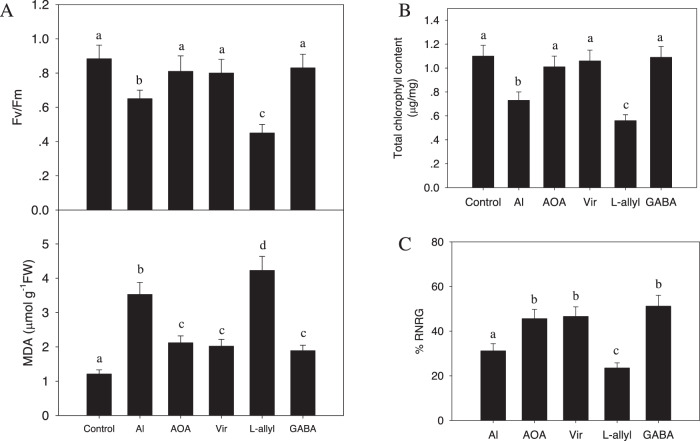
GABA alleviates the growth inhibitory effects of AlCl_3_ stress on hybrid *Liriodendron*. One-week-old hybrid *Liriodendron* seedlings of somatic embryos were subjected to AlCl_3_ stress or AlCl_3_ stress with an additional inhibitor as described above for 3 days. Leaf Fv/Fm, MDA content (**A**), total chlorophyll content (**B**) and relative root growth (**C**) were measured. The experiment was performed in triplicate, and the data represent the mean ± SD of three biological replicates. Different letters indicate statistically significant differences (*p* < 0.05) as determined by Tukey’s multiple comparisons test

Having found that Al stress in hybrid *Liriodendron* leads to increased GABA synthesis, which mediates downstream responses, such as ROS reduction and organic acid exudation, we investigated whether GABA can indeed protect hybrid *Liriodendron* from AlCl_3_-induced damage. We found that exogenous GABA treatment or treatment with AOA and vigabatrin indeed increased the photosynthetic Fv/Fm ratio, increased the chlorophyll level, and reduced the level of MDA in hybrid *Liriodendron* after AlCl_3_ exposure compared with the levels in the control plants (Fig. 7A, B). Consistent with this, L-allylglycine treatment exerted the opposite effects on these parameters (Fig. 7A, B).

Another parameter reflecting plant seedling tolerance to AlCl_3_ stress is the average relative net root growth (RNRG), which compares root length before and after AlCl_3_ treatment^23^. Again, we found that GABA, AOA, or vigabatrin treatment improved root tolerance to AlCl_3_ stress compared to that in control plants, while L-allylglycine reduced the tolerance (Fig. 7C). These data show that GABA signaling acts to increase hybrid *Liriodendron* tolerance to AlCl_3_ by protecting it from the damaging effects of AlCl_3_ toxicity.

The hybrid *Liriodendron* leaf viability/photosynthetic capability was further characterized in response to 30 μM AlCl_3_ and 30 μM AlCl_3_ supplemented with 10 mM GABA. We found that the viability/photosynthetic capability of hybrid *Liriodendron* leaves subjected to AlCl_3_ + GABA after 7 days of treatment was better than that of the AlCl_3_ treatment group (Supplemental Fig. 3). Although the Fv/Fm ratio decreased in the leaves treated with AlCl_3_ + GABA, it was still higher than that in the leaves treated with AlCl_3_. AlCl_3_ + GABA treatment also reduced MDA content, indicating that GABA relieved the membrane lipid oxidative damage. Furthermore, we found that the leaf transpiration rate and stomatal conductance levels, additional parameters that reflect photosynthetic activity, were also increased after AlCl_3_ + GABA treatment compared with AlCl_3_ treatment. Thus, these data suggest that GABA AlCl_3_ positively helps the leaves of hybrid *Liriodendron* resist aluminum stress.

### GABA signaling in response to Al stress may be a conserved pathway in higher plants

**Fig. 8:**
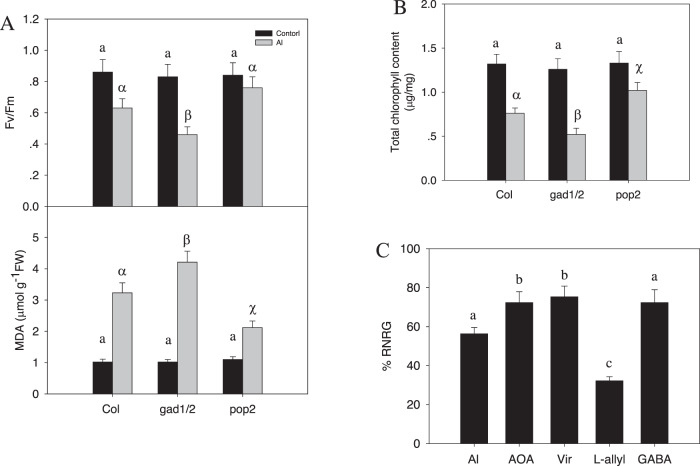
The role of GABA in AlCl_3_ stress signaling is conserved in *Arabidopsis thaliana*. The role of GABA in AlCl_3_ stress signaling is conserved in *Arabidopsis thaliana*. The experiment was performed in triplicate, and the data represent the mean ± SD of three biological replicates. Different letters indicate statistically significant differences (*p* < 0.05) as determined by Tukey’s multiple comparisons test. **A**, **B** Effect of AlCl_3_ on leaf photosynthesis-related Fv/Fm, MDA content, and total chlorophyll content in wild-type Col and in the *gad1/2* and *pop2* mutants. One-week-old seedlings were treated with 30 μM AlCl_3_ for 3 days, and the Fv/Fm ratio and MDA content (**A**), as well as the total chlorophyll content (**B**), were measured. **C** Effect of AlCl_3_, GABA, and GABA biosynthesis inhibitor treatment on relative root elongation. One-week-old seedlings were treated with 30 μM AlCl_3_ or 30 μM AlCl_3_ with different additional chemicals as described above for one week, and the relative root elongation was measured

Our results so far show that GABA enhances hybrid *Liriodendron* tolerance to Al stress, which is consistent with a previous study that showed that wheat species with tolerance to Al contained high levels of GABA^23,24^. In the plant kingdom, GABA has a wide range of regulatory functions in growth, development, abiotic stress responses, and defense^38,39^. Furthermore, GABA can function in different kingdoms because plants, animals, and fungi respond to GABA^38–40^. These results also suggest that GABA signaling, as an Al stress response, is a more widely conserved pathway in higher plants. To investigate this, we turned to the model system *Arabidopsis*. The *Arabidopsis* GABA-T-deficient *pop2-1* mutant showed a high endogenous GABA content, while the *Arabidopsis gad1/2* double mutant contained low levels of GABA^22^. We thus subjected wild-type Col, *pop2*, and *gad1/2* double mutant seedlings to AlCl_3_ stress and then measured their physiological response. We first confirmed that in *Arabidopsis*, Al stress similarly reduced the Fv/Fm ratio and total chlorophyll content and increased the MDA content compared to that in unstressed seedlings (Fig. 8A, B). Consistent with our hypothesis, the *pop2-1* mutant with high endogenous GABA content exhibited reduced Al toxicity, as it showed higher Fv/Fm values and total chlorophyll content, lower MDA levels, and an increased RNRG (Fig. 8C). In contrast, the *gad1/2* mutant with lower GABA levels showed lower Fv/Fm values and total chlorophyll content, higher MDA levels, and a lower RNRG. These findings indicate the critical role of GABA in enhancing plant tolerance to Al stress; furthermore, this function is possibly conserved across widely divergent plant species.

## Discussion

Most woody plants grow on acidic soil and show tolerance to Al stress, but the mechanism underlying this tolerance remains unknown. To address this question, we used hybrid *Liriodendron*, a relative of *L. chinense* that has been sequenced and developed as a model system for woody plants in our lab, because of its ecological and economic value for wood cultivation^25^. We opted to use a proteomics approach to identify genetic pathways involved in Al stress resistance. We found that a large variety of pathways respond to Al toxicity and detected changes in auxin, BR, and GA biosynthesis pathways. We also detected changes in transcription factors such as WRKY and basic HLH, which are involved in Al stress tolerance in *Arabidopsis*; for example, *Arabidopsis* WRKY46 plays a role in Al tolerance^16^. Proteins involved in protein degradation and methylation status also regulate Al resistance in *Arabidopsis*^41,42^. Based on these findings, we propose that hybrid *Liriodendron* adopts a wide variety of strategies to control its Al stress response.

More specifically, we found that several metabolic enzymes involved in the synthesis of the signaling molecule GABA, including GAD (Lchi33118, Lchi05759, and succinate-semialdehyde dehydrogenase (Lchi21261), showed an increased expression level under Al stress. Accumulated evidence demonstrates that GABA has a physiological function in plant growth, development and defense responses^19–21^. Consistent with this finding, we found that Al stress indeed induced an increase in GAD1 activity and that GABA production by GAD1 was also increased after Al stress. Inhibiting the GAD1 enzyme activity by using the specific inhibitor L-allylglycine obviously reduced Al-induced GABA generation, supporting the hypothesis that Al-induced GABA biosynthesis depends on GAD activity. In plants, GABA is dynamically degraded by GABA-Tase VOA and Vir. We also treated hybrid *Liriodendron* seedlings with VOA or Vir and found that the treatment further enhanced Al-induced GABA biosynthesis by suppressing GABA-Tase activity and compromising GABA degradation. The essential role of the GABA shunt in preventing ROS generation and cell death during UV or heat stress has been reported. Here, our iTRAQ data showed that Al stress upregulated the protein levels of several antioxidant enzymes, including CAT and SOD. Rapid accumulation of ROS, mainly H_2_O_2_ and O^2−^, and an increase in antioxidant enzyme activity were observed after exposure to Al stress. Furthermore, we found that supplementation with GABA or enhancement of endogenous GABA levels by AOA or Vir enhanced Al-induced antioxidant enzyme activity and reduced ROS accumulation, while suppressing GAD1 enzyme activity by using L-allylglycine compromised GABA levels and reduced antioxidant enzyme activities, leading to increased ROS accumulation. A previous study showed that the GABA shunt was involved in ROS metabolism during UV or salt stress. Our finding here is in agreement with this finding and demonstrated that GABA efficiently increased antioxidant enzyme activities for scavenging ROS during Al stress. Our iTRAQ results also showed that Al stress induced the protein accumulation of P5CS, which is the key enzyme for proline biosynthesis. Proline also accumulated at high levels in plants under environmental stress^43^. Environmental stress induces the generation of proline to balance cellular osmotic pressure^33,34,43^, and proline has been shown to perform multiple antioxidant functions^44^. Here, we found that Al stress induced the biosynthesis of proline. Additional GABA or GABA-Tase inhibitor treatment further increased Al-induced proline biosynthesis, while suppressing GABA biosynthesis by L-allylglycine also reduced Al-induced proline biosynthesis. These data suggest that GABA also controls Al-induced proline biosynthesis, which may be used for multiple antioxidant responses under Al stress. Unlike drought stress, proline, when accumulated at high levels, is believed to be not only an osmolyte but also a signaling molecule that provides defense against oxidative damage.

Secretion of organic acids, including malate and citrate, to chelate Al plays an essential role in plant tolerance to Al stress^4^. ALMT facilitates malate efflux, while MATEs facilitate citrate efflux^10^. Our iTRAQ results demonstrated that Al stress triggered the upregulation of MATE1 and MATE2. RT-qPCR results also showed that Al stress increased the transcriptional level of *MATE1/2*, suggesting the possible role of MATE-mediated citrate in Al tolerance. Consistent with this finding, we found that Al stress indeed induced an increase in citrate in hybrid *Liriodendron* roots, and the citrate efflux capability also increased after Al stress. These results suggest that citrate efflux chelates Al enhance hybrid *Liriodendron* tolerance to Al stress. A previous study demonstrated that GABA regulates ALMT for malate efflux. Here, we also found that additional GABA, AOA, or Vir treatment increased the transcription of *MATE1/2* and the efflux of citrate, which may explain why GABA signaling enhances hybrid *Liriodendron* tolerance to Al stress. In contrast, suppressing GABA biosynthesis by using L-allylglycine compromised Al-induced transcription of *MATE1/2* and decreased citrate efflux. These data further support the critical role of GABA signaling in promoting citrate efflux for Al tolerance. Moreover, we found that GABA signaling also enhanced Al-induced malate efflux. In wheat roots, Al-activated malate transport also facilitates GABA transport; thus, whether or how GABA signaling controls malate or citrate efflux through ALMT or MATE1/2 needs further investigation.

In conclusion, our proteomics approach using quantitative iTRAQ technology to analyze the differential expression of proteins in hybrid *Liriodendron* leaves subjected to Al stress found that hybrid *Liriodendron* likely adopts multiple strategies to enhance its Al stress tolerance: changes in metabolism, sugar and proline biosynthesis, differential transcription factor expression, cell autophagy and ubiquitin-dependent protein degradation to anion transport. Through further study, we revealed a novel role for the nonclassic amino acid GABA, which controls multiple aspects of the Al stress response in not only hybrid *Liriodendron* but also *Arabidopsis*. Therefore, our results help to further explain how plants combat Al toxicity, and these findings should aid in the broad application of gene engineering in hybrid *Liriodendron* and other plants to improve their Al tolerance.

## Supplementary information

Supplemental Figures

Supplemental Table 1

Supplemental Table 2
