# Supplementary material for: The role of γ-aminobutyric acid in aluminum stress tolerance in a woody plant, Liriodendron chinense × tulipifera
Source: Hortic Res. 2021 Apr 1;8:80. doi: 10.1038/s41438-021-00517-y (PMC8012378; doi:10.1038/s41438-021-00517-y)

Supplemental Figure 1

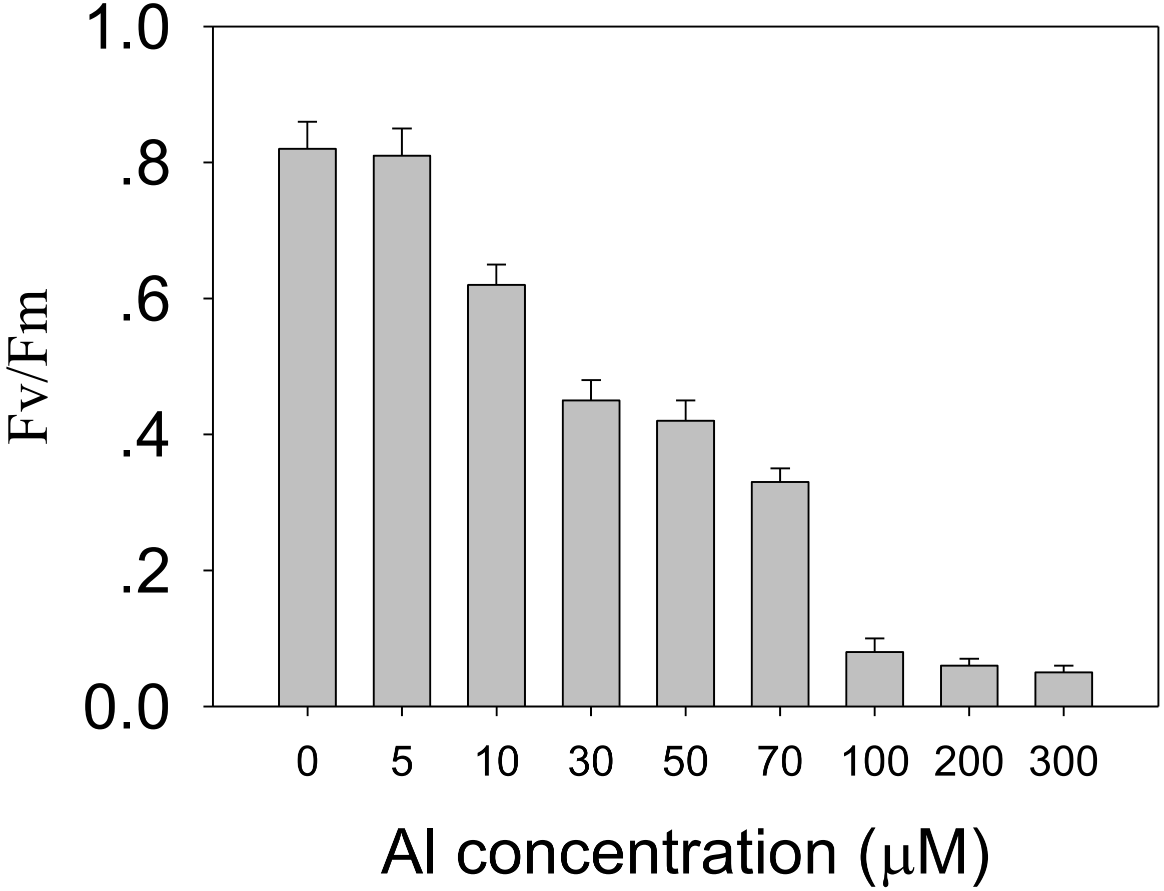

Supplemental Figure 2

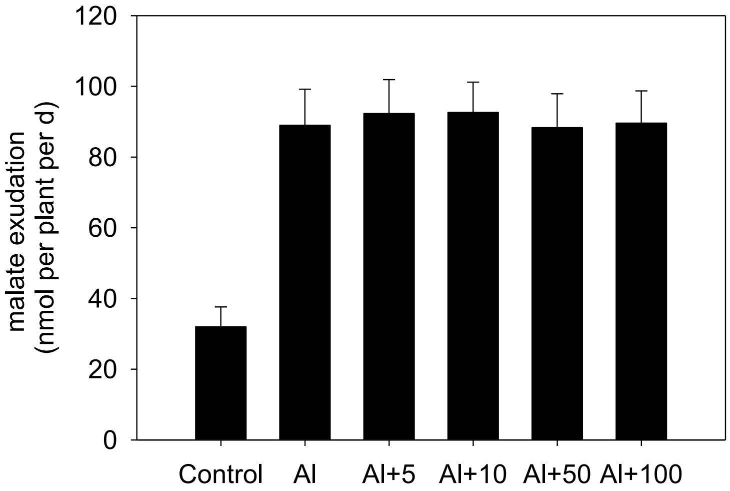

Supplemental Figure 3

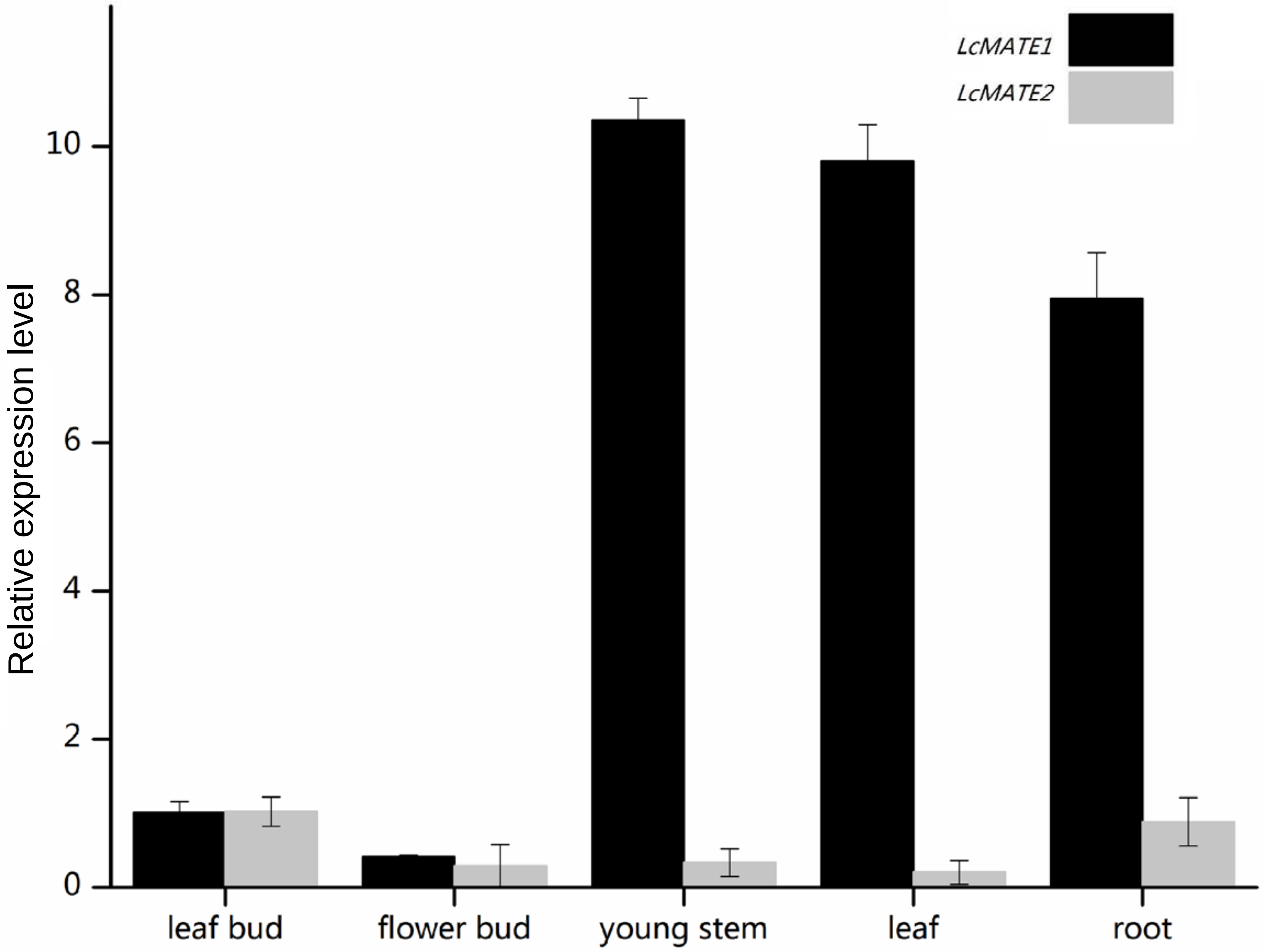

Supplemental Figure 4

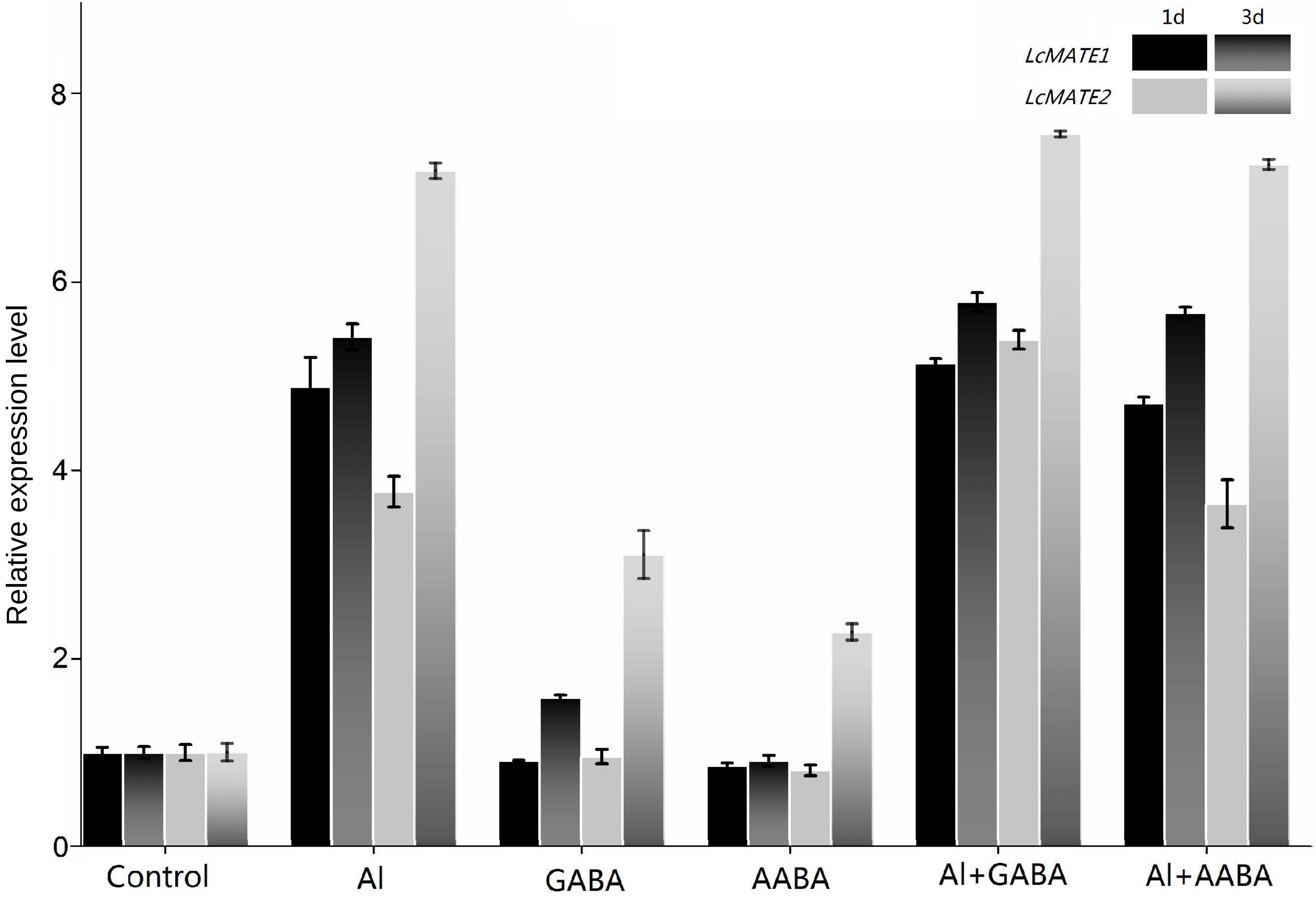

Supplemental Figure 5

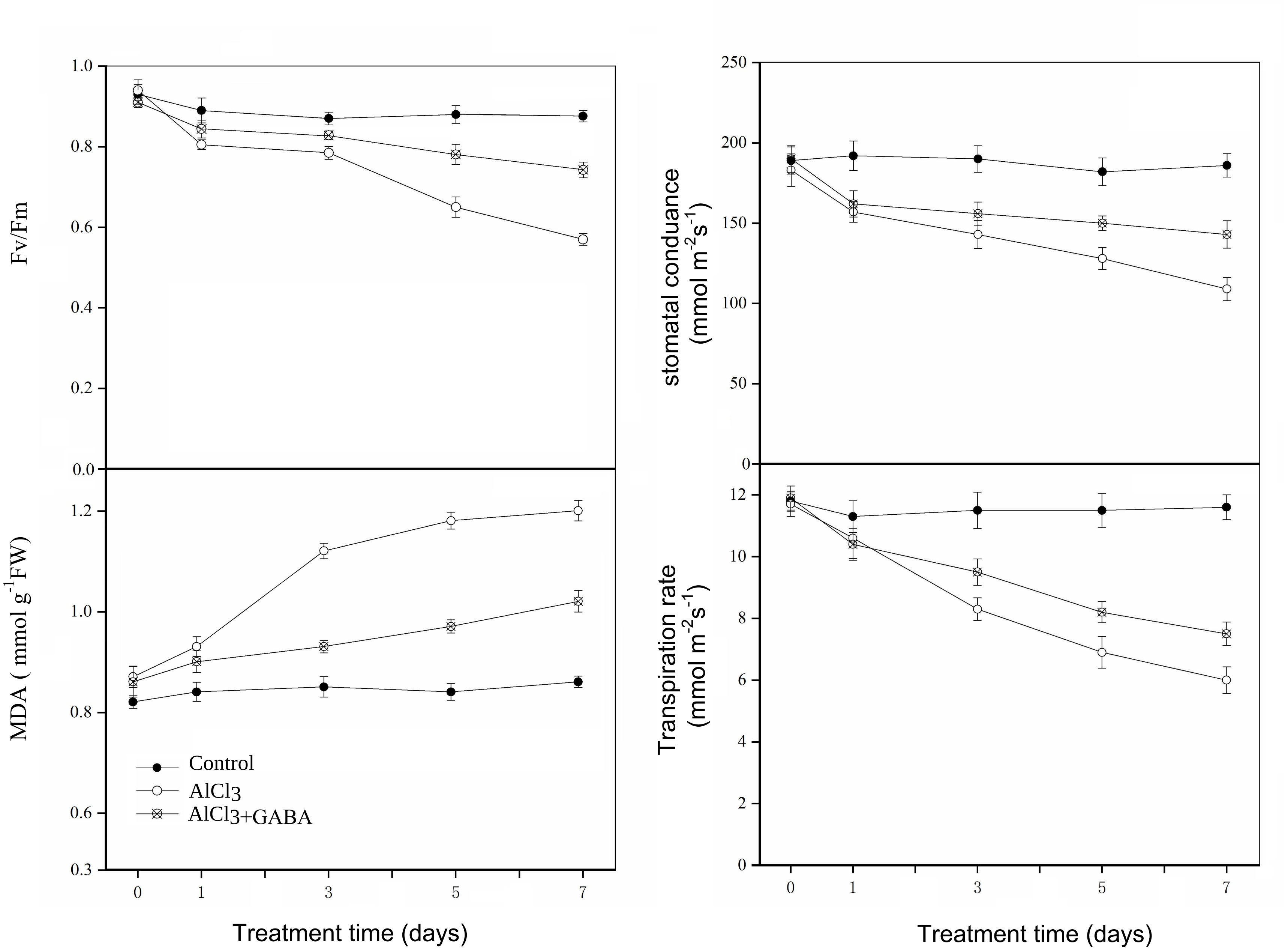

Supplement: Supplementary file 1 — Supplemental Figures [file 41438_2021_517_MOESM1_ESM.pdf]
